# Neighboring trees regulate the root‐associated pathogenic fungi on the host plant in a subtropical forest

**DOI:** 10.1002/ece3.6094

**Published:** 2020-04-23

**Authors:** Keke Cheng, Shixiao Yu

**Affiliations:** ^1^ Department of Ecology School of Life Sciences/State Key Laboratory of Biocontrol Sun Yat‐sen University Guangzhou China

**Keywords:** host specificity, Janzen–Connell hypothesis, neighbor influences, phytopathogenic fungi, root‐associated fungi, subtropical forest

## Abstract

Root‐associated fungi and host‐specific pathogens are major determinants of species coexistence in forests. Phylogenetically related neighboring trees can strongly affect the fungal community structure of the host plant, which, in turn, will affect the ecological processes. Unfortunately, our understanding of the factors influencing fungal community composition in forests is still limited. In particular, investigation of the relationship between the phytopathogenic fungal community and neighboring trees is incomplete. In the current study, we tested the host specificity of members of the root‐associated fungal community collected from seven tree species and determined the influence of neighboring trees and habitat variation on the composition of the phytopathogenic fungal community of the focal plant in a subtropical evergreen forest. Using high‐throughput sequencing data with respect to the internal transcribed spacer (ITS) region, we characterized the community composition of the root‐associated fungi and found significant differences with respect to fungal groups among the seven tree species. The density of conspecific neighboring trees had a significantly positive influence on the relative abundance of phytopathogens, especially host‐specific pathogens, while the heterospecific neighbor density had a significant negative impact on the species richness of host‐specific pathogens, as well as phytopathogens. Our work provides evidence that the root‐associated phytopathogenic fungi of a host plant depend greatly on the tree neighbors of the host plant.

## Graphical abstract

The Janzen–Connell hypothesis proposes that plant interactions with host‐specific antagonists can maintain high diversity in tropical forest. However, there is hardly any knowledge about the relationship between the pathogenic fungal community and neighboring trees. To address this knowledge gap, we characterized the community composition of the root‐associated fungi for seven tree species in a subtropical evergreen forest by using high‐throughput sequencing data with respect to the internal transcribed spacer (ITS) region. We found significant differences with respect to fungal groups among the seven tree species, and the density of neighboring trees had a significantly positive influence on the relative abundance of host‐specific pathogens.

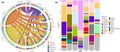

## 1 INTRODUCTION

It has been challenging for ecologists to understand the mechanisms by which abundant species coexist in plant communities for decades. An influential explanation for this maintenance of species diversity is the Janzen–Connell hypothesis (Connell, 1971; Janzen, 1970), which proposes that host‐specific natural enemies, such as fungal pathogens and herbivores, spread from adult trees to their nearby offspring in a density‐dependent trend, which leaves a vacant space for the establishment of other species. Recently, numerous experimental and phenomenological investigations have provided evidence to support this hypothesis in tropical and temperate forests (Comita et al., 2014; Gilbert, Harms, Hamill, & Hubbell, 2001; Packer & Clay, 2000; Peters, 2003). Moreover, an increasing number of researchers have reported the potential roles of fungal phytopathogens, which have been interpreted as a key element to species coexistence (Bagchi et al., 2010; Bradley, Gilbert, & Martiny, 2008; Liu, Etienne, Liang, Wang, & Yu, 2015).

In natural forests, plant roots are colonized by a large number of fungi (Vandenkoornhuyse, Baldauf, Leyval, Straczek, & Young, 2002), with a species‐specific fungal community developing in their rhizosphere in response to exudates and other products secreted by the roots (Broeckling, Broz, Bergelson, Manter, & Vivanco, 2008; Hartmann, Schmid, Tuinen, & Berg, 2009). As described in the Janzen–Connell hypothesis, host‐specific fungal phytopathogens that accumulate around the parent trees affect the subsequent growth and performance of the resulting conspecific seedlings (Bever, Mangan, & Alexander, 2015; Klironomos, 2002). However, our knowledge of host‐specific pathogens, as well as the identity of the plant root‐associated fungi or the fungal community structure, is still scarce.

At the local scale, various factors are known to affect the composition of the root‐associated fungal community. Primarily, the fungal community associated with the root is strongly influenced by host characteristics such as host genotype (Bálint et al., 2013), root carbohydrates (Hadacek & Kraus, 2002), and physical or chemical defenses (Saunders & Kohn, 2009). When multiple plant hosts are present, neighboring plants can have a significant impact on the fungal community composition (Hausmann & Hawkes, 2009; Hubert & Gehring, 2008) with high plant diversity reduces the rate of spread of specialist pathogen (Hunter & Aarssen, 1988; Rottstock, Joshi, Kummer, & Fischer, 2014). Moreover, observational research has pointed out that the focal plant's performance and maintenance may be inhibited by its phylogenetically related heterospecific neighbors, the latter might be infected by phytopathogens that can transmit among phylogenetically and functionally similar species (Lebrija‐Trejos, Wright, Hernández, & Reich, 2014; Liu et al., 2012; Parker et al., 2015; Webb, Gilbert, & Donoghue, 2006).

In addition, extensive research has demonstrated that the fungal community and the plant host depend on environmental factors, such as topography, and soil properties including organic matter content, soil pH, and nutrient availability (Conn & Dighton, 2000; Lilleskov, Fahey, & Lovett, 2001; Messaoud & Houle, 2006; Summerbell, 2005). Under certain conditions, root‐associated fungal communities may exhibit habitat specificity (Fujimura & Egger, 2012). To date, many studies have been undertaken to examine the effects of environmental variables and neighboring plants on individual host plant survival. However, only a few such studies have reported on the interaction between host plants and their root‐associated fungal community, and the influence of habitat variation and neighboring plants, taking phylogenetic distance of neighboring plants into consideration.

Plant–fungus interactions and host‐specific pathogens have been recognized to be important determinants of plant community assembly and ecosystem function (Benítez, Hersh, Vilgalys, & Clark, 2013; Bever et al., 2015). However, identification of the processes underlying the composition of fungal communities associated with plant roots, as well as the generality of host specificity in root pathogens, remains speculative. Meanwhile, understanding the relative importance of these influencing factors, such as habitat variation and phylogenetically distinct neighboring plants, is central to resolving plant–pathogen feedback, a phenomenon remaining poorly understood in natural ecosystems.

In the present study, we explored the relationship between the root‐associated fungal community of the host species and the impacts of neighboring trees and environmental variation in a subtropical forest. We collected seedling roots from seven plant species in a broad‐leaved evergreen forest in southern China. Based on second‐generation sequencing data, we explored the composition of the root‐associated fungal community on different host tree species, categorized fungi to potential pathogen that infect plants, and addressed the following questions: (a) Is the composition of the root‐associated fungal and phytopathogenic fungal communities controlled by the host plant?; (b) Does the variation in neighboring plants and habitat influence the composition of the total and phytopathogenic fungal communities?; and (c) How do the densities and phylogenetic relatedness of the neighboring trees and habitat variation affect the abundance and richness of phytopathogens, especially host‐specific pathogens?

## 2 MATERIALS AND METHODS

### 2.1 Study sites and selected species

The fieldwork was conducted in Heishiding Nature Reserve (Guangdong Province, southern China; 111°53′h, 23°27′N, 150–927 m above sea level), where the study area consists of approximately 4,200 ha covered with subtropical evergreen broad‐leaved monsoon forest (Yu, Li, Wang, & Zhou, 2000). Precipitation in Heishiding is variable and influenced by two seasons: a humid season from April to September and a dry season from October to March, with an average annual precipitation of 1744 mm. Temperatures vary throughout the year, with summer maxima exceeding 28.4°C in July and winter temperatures falling to as low as 10.6°C in January.

Seven evergreen broad‐leaved tree species were chosen as the focal plants in this study: *Castanopsis fabri* Hance (Fagaceae), *Castanopsis fissa* Rehd. et Wils (Fagaceae), *Cyclobalanopsis fleuryi* (Hick. et A. Camus) Chun ex Q. F. Zheng (Fagaceae), *Engelhardia fenzelii* Merr. (Juglandaceae), *Artocarpus styracifolius* Pierre (Moraceae), *Canarium album* (Lour.) Raeusch. (Burseraceae), and *Cryptocarya concinna* Hance (Lauraceae). Each species is common in the study area, and their seeds are gravity‐dispersed, with the exception of *E. fenzelii*, seeds of which are wind‐dispersed. *C. concinna* is a pioneer species, whereas *Castanopsis* is a heliophilous genus and *C. album* is a late‐successional and shade‐tolerant species. All seven species are evergreen trees widespread throughout temperate and subtropical forests in southern China and are keystone species in Heishiding Forest (Li, Yu, Lian, Zhou, & Wang, 2000).

### 2.2 Field sampling

For each target species, five adult trees, each having more than four conspecific seedlings at a distance of 0–2 m, were randomly selected. The root systems of three to six conspecific seedlings associated with each focal species were collected as intact as possible. The whole root system of each plant was placed in a Ziploc resealable plastic bag, which was placed on ice immediately in the field. Each root sample was washed with sterilized deionized water to remove adhering soil particles and frozen at −20°C on the day of collection. Samples were then transported to the laboratory under dry ice for subsequent microbial community analysis within 1 week of collection. All the field sampling was carried out in August 2017.

### 2.3 Environmental factors and soil properties

Environmental factors, such as canopy openness, slope, and aspect, were collected for each focal adult parent tree. Hemispherical photographs were taken in four directions around the adult tree with a leveled Nikon COOLPIX 4500 camera body and Nikon FC‐E8 fisheye converter lens to determine canopy openness. Slope and aspect were recorded using a digital gradiometer and a compass, respectively.

Soil samples (collected at 0–10 cm depth), excluding the litter layer, were also collected around the adult tree randomly in four directions by auger boring (diameter with 50 mm) and placed in a Ziploc bag and thoroughly mixed. The soil was loosely covered to air‐dry and sieved through a 2‐mm sieve to homogenize and to remove coarse fragments. Subsequent soil analyses were performed using standard methods (Gregorich & Carter, 2007). Briefly, soil pH was determined on a soil: water (1:5 w/v) suspension using a digital pH meter, soil organic carbon (OC) concentration was determined by the dichromate–sulfuric acid oxidation method, total nitrogen (TN) concentration was measured by the Kjeldahl method, and available nitrogen (AN) concentration determination was conducted by the alkali diffusion method. Total phosphorus (TP) concentration was tested by the sulfuric acid–perchloric acid digestion method and available phosphate (AP) concentration was determined following the molybdenum stibium anti‐spectrophotography method. Total potassium (TK) concentration was analyzed following digestion with hydrofluoric acid and perchloric acid, whereas available potassium (AK) concentration was determined by ammonium acetate extraction and quantification by the colorimetric method.

### 2.4 Vegetation survey

In order to investigate the effect of local neighboring trees on the root‐associated fungal community of each focal tree, a 40 × 40 quadrat (20‐m radius circle) was established centered on the tree. Details of each tree with a diameter at breast height (dbh) >1 cm were recorded, including species names, height, dbh, and position relative to the focal tree at each of our study plots. For each focal adult tree, we determined the sum of the basal area of both conspecific and heterospecific individuals of all trees within 20 m of the focal tree and expressed them as conspecific neighbor density and heterospecific neighbor density, respectively (Metz, Sousa, & Valencia, 2010; Stoll & Newbery, 2005). To quantify the phylogenetic relatedness between the focal tree and their neighboring plants, we constructed a phylogenetic tree using Phylomatic (Webb & Donoghue, 2005) based on the angiosperm phylogeny group (APG) III backbone phylogeny (http://phylodiversity.net/phylomatic/) (Angiosperm Phylogeny Group, 2009). Then, we added the branch lengths to the phylogenetic tree using the BLADJ algorithm in Phylocom 4.2 (Webb, Ackerly, & Kembel, 2008) and match the node age from Wikström, Savolainen, and Chase (2001). Finally, we calculated the phylogenetic distance between the focal tree and each of its neighboring trees using the Phydist function of Phylocom (Webb et al., 2008) and compared the result with Yang et al. (2014).

### 2.5 Molecular characterization of root‐associated fungi

Total genomic DNA was extracted from each plant root sample using Plant Genomic DNA kit (Tiangen Biotech), and the DNA concentration was quantified by Qubit 3.0 fluorometer (Invitrogen). After extraction, 20–30 ng plant root DNA was used to amplify the fragments of the internal transcribed spacer (ITS) sequence by ITS1 (5'‐GTGAATCATCGARTC‐3') and ITS4 (5'‐TCCTCCGCTTATTGAT‐3') primers which were proposed as the tagged fungal specific primers (Schoch et al., 2012). Additionally, the primers ITS1 and ITS4 contained the complementary adaptor sequences for the Illumina MiSeq platform.

PCRs were performed in triplicate 25 μl mixture containing 20 ng of template DNA, 1 μl of each primer (ITS1, ITS4), 2 μl of deoxynucleoside triphosphate (dNTPs), 2.5 μl of FastPfu Buffer, and 0.5 μl of FastPfu Polymerase (TransGen Biotech). The PCR amplification profile consisted of an initial denaturation of 5 min at 94°C, followed by 25 cycles of 30 s at 94°C, 30 s at 57°C, and 30 s at 72°C, and then a final additional extension step for 5 min at 72°C. PCR amplification products were detected by agarose gel electrophoresis and purified using AxyPrep DNA Gel Extraction kit (Axygen Biosciences). The concentration of purified products was validated by Qubit 3.0 fluorometer and diluted to 10 nM. The amplicon libraries were sequenced using 2 × 300 bp paired‐end configuration by Illumina MiSeq platform following standard manufacturer's protocols (Illumina). Image analysis and base calling were conducted by the MiSeq Control Software (MCS) embedded in the MiSeq instrument.

Data generated by sequencing were analyzed by QIIME data analysis package (Caporaso et al., 2010). Raw sequences were demultiplexed and trimmed to remove the short and low‐quality sequences with the reads length less than 200 bp, or the reads containing ambiguous bases, or the reads with an average quality score <20. The forward and reverse reads were joined and assigned to samples based on barcode, and then, the sequences were trimmed by removing the barcode and primer sequence. The chimeric sequences were identified with the reference database and removed using UCHIME algorithm (Edgar, Haas, Clemente, Quince, & Knight, 2011). The effective sequences were grouped to operational taxonomic units (OTUs) using the clustering program VSEARCH (Edgar, 2010) against the UNITE ITS database (https://unite.ut.ee/) at 97% sequence identity similarity, and a representative sequence was then assigned for each OTU by selecting the most abundant sequence. All the singleton OTUs were retained. Subsequently, taxonomic category of all OTUs was determined using The Ribosomal Database Program (RDP) classifier (Wang, Garrity, Tiedje, & Cole, 2007) trained on the UNITE ITS database (Abarenkov et al., 2010) with a confidence threshold of 80%. Sequences were rarefied based on the minimum sample to eliminate the unequal sequencing depth between samples prior to statistics analyses. All sequencing runs and data filtering were conducted at GENEWIZ, Inc. (Suzhou, China).

All analyzed fungal OTUs were assigned into several functional characterization groups by FUNGuild program and database (Nguyen et al., 2016). In our study, the comparison was achieved between total fungal communities and phytopathogenic fungal communities, which were separated and defined as potential pathogen can infect plants by FUNGuild (Data S2).

### 2.6 Statistical analysis

Fungal community composition was carried out by nonmetric multidimensional scaling (NMDS) (Kruskal, 1964), demonstrated by the “metaMDS” function in the R package vegan (Oksanen et al., 2013) based on the Bray–Curtis distance metric. Comparison among plant species was calculated via the permutation‐based multivariate analysis of variance (PERMANOVA) (Anderson, 2001; McArdle & Anderson, 2001) with 999 permutations by the “adonis” function. To examine whether this plant–fungal community association was a nonrandom network, the H2’ index, a measure of network‐level specialization, was estimated using the “H2fun” function in the bipartite package (Dormann, Fründ, Blüthgen, & Gruber, 2009).

To illustrate impacts of host species, neighbor plants and environmental factors on the fungal community, the abundance of fungal OTUs of the seedlings around the same focal adult tree was averaged in subsequent analytical methods. Mantel test with the “mantel” function in vegan package (Oksanen et al., 2013) served to explore the correlations between the dissimilarity of fungal community and host species phylogeny. Variation partitioning analysis was performed by dissecting any independent and combined effects in plant hosts, neighboring plants, and environmental variations on the fungal community structure using the “varpart” function. To investigate possible relationship between fungal community and either abiotic or biotic factors, canonical correspondence analysis (CCA) was implemented by “adonis” function with 999 permutations.

In all analytical methods mentioned above, the OTU abundance data were performed on total fungi level as well as phytopathogenic fungi level separately.

Additionally, we separated phytopathogenic fungi into specialist pathogen and generalist pathogen. The pathogenic OTUs detected in only one single plant species was identified as host‐specific pathogens for each of the seven tree species studied. Otherwise was identified as generalist pathogens. For all pathogens and specialist pathogens, the species richness and relative abundance were calculated and used in latter analyses. Generalized linear models (GLM) were used to determine whether habitat variables or neighboring plants had a significant impact on the relative abundance and species richness of phytopathogens, especially on host‐specific pathogens. Moreover, a stepwise random selection procedure was used to find the most significant factor based on Akaike's information criterion (Akaike, 1974; Wagenmakers & Farrell, 2004).

All analyses were carried out in the R environment (Team, 2018) using one or more of the following packages: “vegan” (Oksanen et al., 2013), “ecodist” (Goslee & Urban, 2007), “bipartite” (Dormann et al., 2009), “Lme4” (Bates, Maechler, Bolker, & Walker, 2014), and “ggplot2” (Wickham, 2016).

## 3 RESULTS

### 3.1 Specialization in root‐associated fungi networks

**Figure 1:**
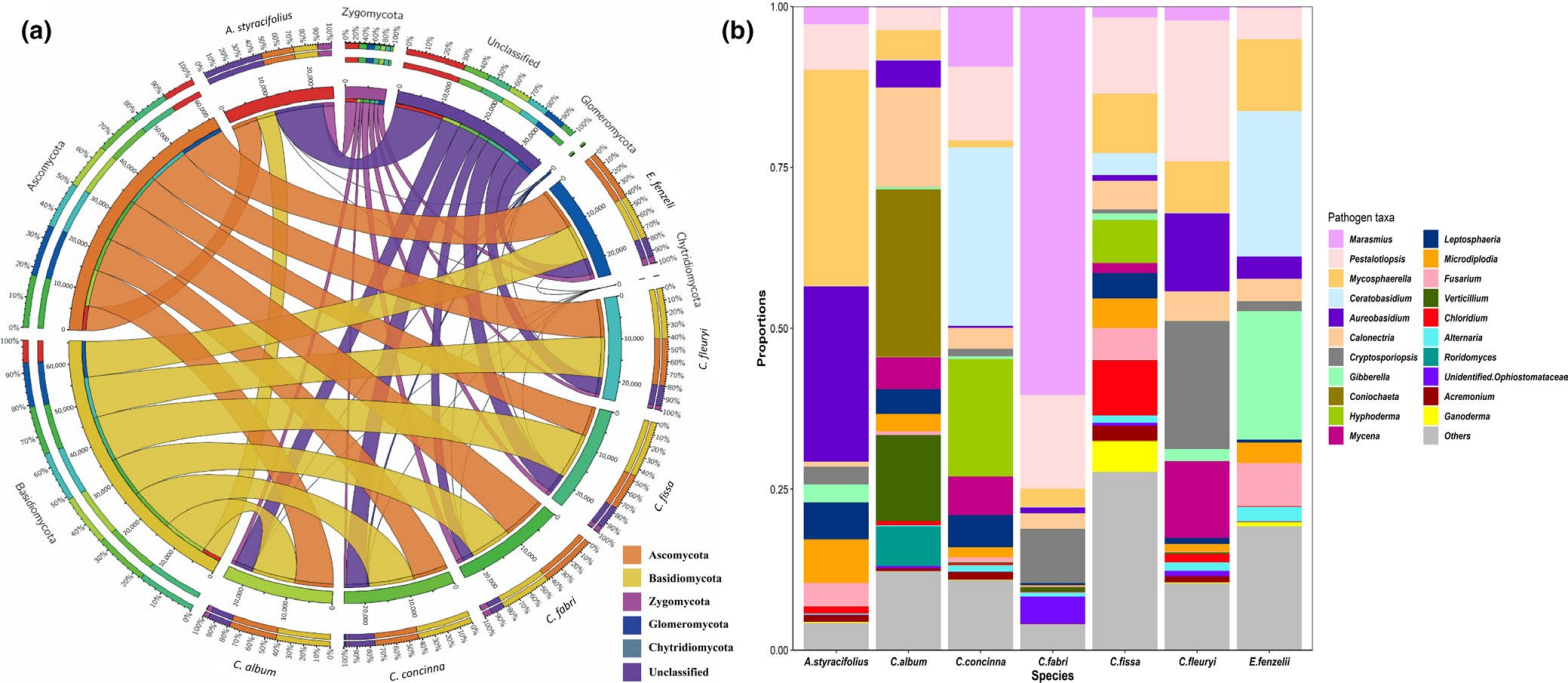
(a) Circos plot of the phylum‐level taxonomic composition of fungi in seven plant species. The width of the bars between a given phylum and a given plant species indicates their relative abundance to the other phyla described. (b) Relative abundance of phytopathogenic fungi at genus level in seven plant species

In total, 14,269,846 sequence reads were generated from 174 root samples. After filtering, 10,712,352 sequence reads were obtained with an average of 61,565 (*SD* = 27,459) sequences per sample. These sequences were clustered into 4,323 OTUs, representing 70 orders, 161 families, and 351 genera. At the phylum level, most of these fungal OTUs belong to Basidiomycota (38.41 ± 25.01%) or Ascomycota (35.29 ± 20.11%). The Zygomycota comprised 5.18 ± 9.05%, whereas the Glomeromycota (0.34 ± 0.8%) and Chytridiomycota (0.03 ± 0.08%) each contributed <1% of all fungal OTUs (Figure 1a). On the basis of the functional composition of fungal communities, 230 fungal OTUs were categorized as plant–pathogen, based on their taxonomy. The most abundant genera among the pathogenic fungi were *Pestalotiopsis* (14.84 ± 23.11%), *Mycosphaerella* (9.72 ± 16.74%), and *Calonectria* (9.41 ± 20.08%) (Figure 1b).

**Figure 2:**
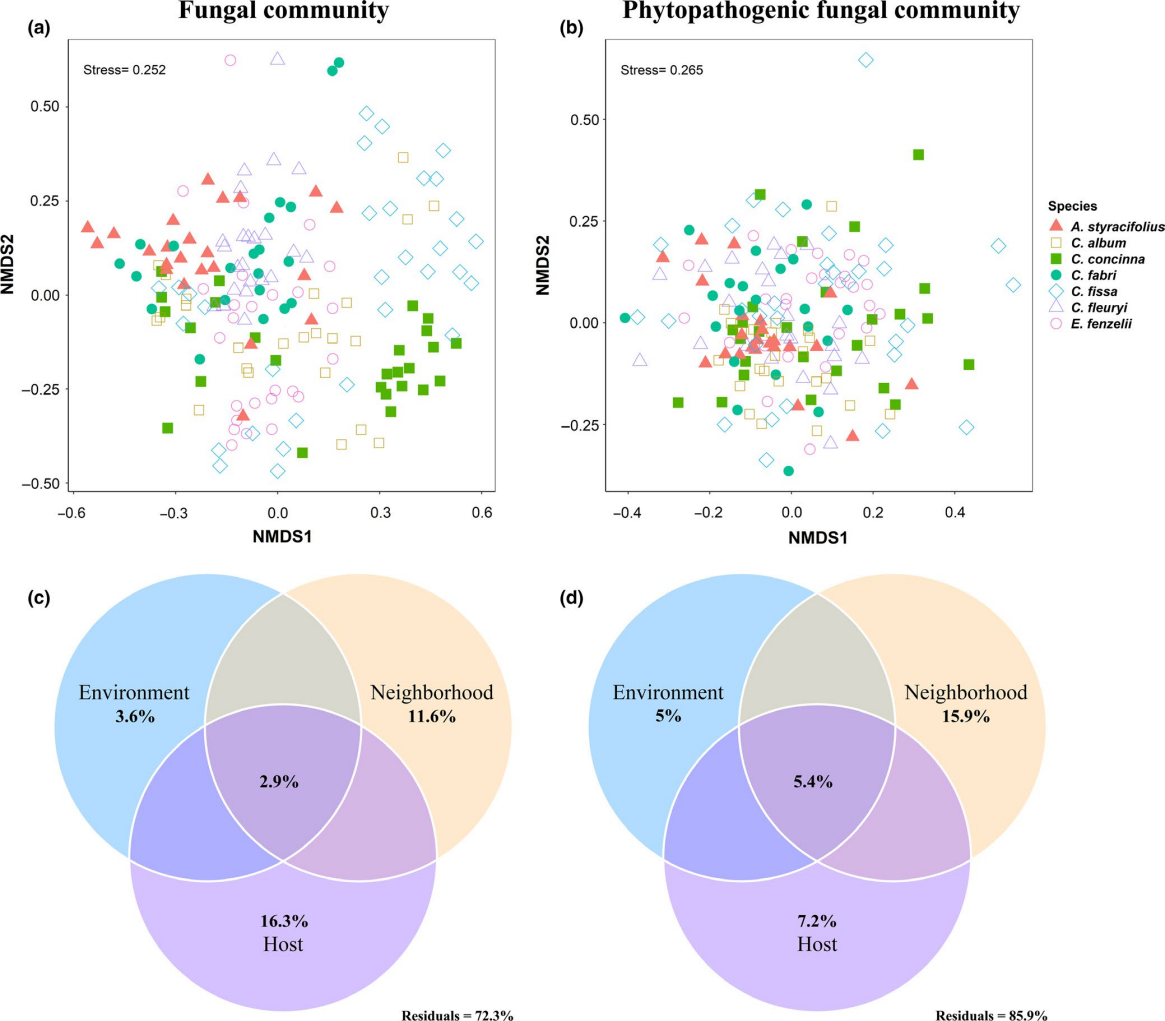
(a and b) Community ordinations using nonmetric multidimensional scaling (NMDS) for the root‐associated fungal community composition (a, fungi, stress = 0.252) and phytopathogenic root‐associated fungal community composition (b, phytopathogenic fungi, stress = 0.265) based on Bray–Curtis distance in the 174 root samples across the seven plant species. (c and d) Variation partitioning analysis showing the pure and shared effects of host plant, neighbor plant, and environmental factors on the fungal community (c) and phytopathogenic fungal community (d). Values in the circles indicate the proportion of explained variation

The compositional structure of the fungal communities differed significantly based on the various plant species (PERMANOVA, *F* = 4.913, *df* = 6, *p* < .001; Figure 2a). More importantly, host preference was also observed for the assemblages of phytopathogens (*F* = 1.932, *df* = 6, *p* < .001; Figure 2b).

Meanwhile, variation partitioning analysis showed host plant independently accounted for 16.3% of the variation in fungal community, and 11.6% was associated with neighboring plants (Figure 2c). However, neighboring plants were the main factor influencing the phytopathogenic fungal community rather than host plant and environmental predictors (Figure 2d). Environmental factors accounted a much smaller amount of variation in both fungal (3.6%) and phytopathogenic fungal (5%) community. Host plant, neighboring plants, environmental variations, and their interactions together explained 27.7% and 14.1% of the total variation in fungal and phytopathogenic fungal community, respectively (Figure 2c,d).

The Mantel test showed that both fungal and phytopathogenic fungal community composition were significantly correlated with host phylogenetic distance (fungi: *r* = .181, *p* = .001; phytopathogenic fungi: *r* = .058, *p* = .027). In addition, we found that the network‐level specialization index H2' was significantly higher than the null model values for both root‐associated fungal networks and phytopathogenic fungal networks, indicating that fungal community structure was more specialized with the host than random (Table S1).

In summary, analyses of the community composition of both root‐associated fungi and phytopathogenic fungi from the seven species located in the subtropical forest suggested that host specificity existed in fungal communities.

### 3.2 Relationships between fungal community composition and phylogenetically related neighbors and habitat variation

**Figure 3:**
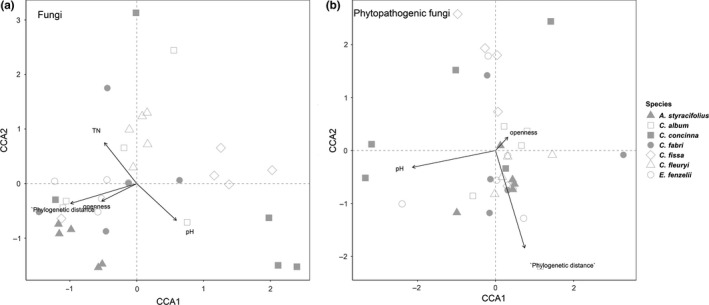
Canonical correspondence analysis (CCA) shows the relationships between the significant variables and (a) the root‐associated fungal community, (b) the phytopathogenic root‐associated fungal community. The arrows indicate effect factors, while the length of each arrow represents the strength of the relationship between the habitat variable and the distribution of fungal community

The CCA plot explained 61.04% (all fungi: axis 1 = 33.67%, axis 2 = 27.37%; Figure 3a) and 73.02% (phytopathogenic fungi: axis 1 = 40.97%, axis 2 = 32.05%; Figure 3b) of the observed variation in fungal community and phytopathogenic fungal community, respectively.

According to PERMANOVA (Table S2), soil pH (*r^2^* = .039, *p* = .049) and total nitrogen (*r^2^* = .044, *p* = .014) of the soil properties, and canopy openness (*r^2^* = .061, *p* = .003) of the environmental factors and phylogenetic distance of neighbors (*r^2^* = .053, *p* = .007) showed significant correlations with the change of the fungal community, whereas the phytopathogenic fungal community was significantly correlated with only canopy openness (*r^2^* = .047, *p* = .028) and phylogenetic distance of neighbors (*r^2^* = .042, *p* = .038). However, of all the soil properties tested, only soil pH had a weak effect on the change of the phytopathogenic fungal community.

In summary, phylogenetic distance of the neighboring trees from the focal tree and canopy openness was identified as important factors influencing the fungal community, with respect to both total fungi and phytopathogenic fungi.

### 3.3 Influences of neighboring plants and habitat variation on relative abundance and species richness of host‐specific pathogens

**Figure 4:**
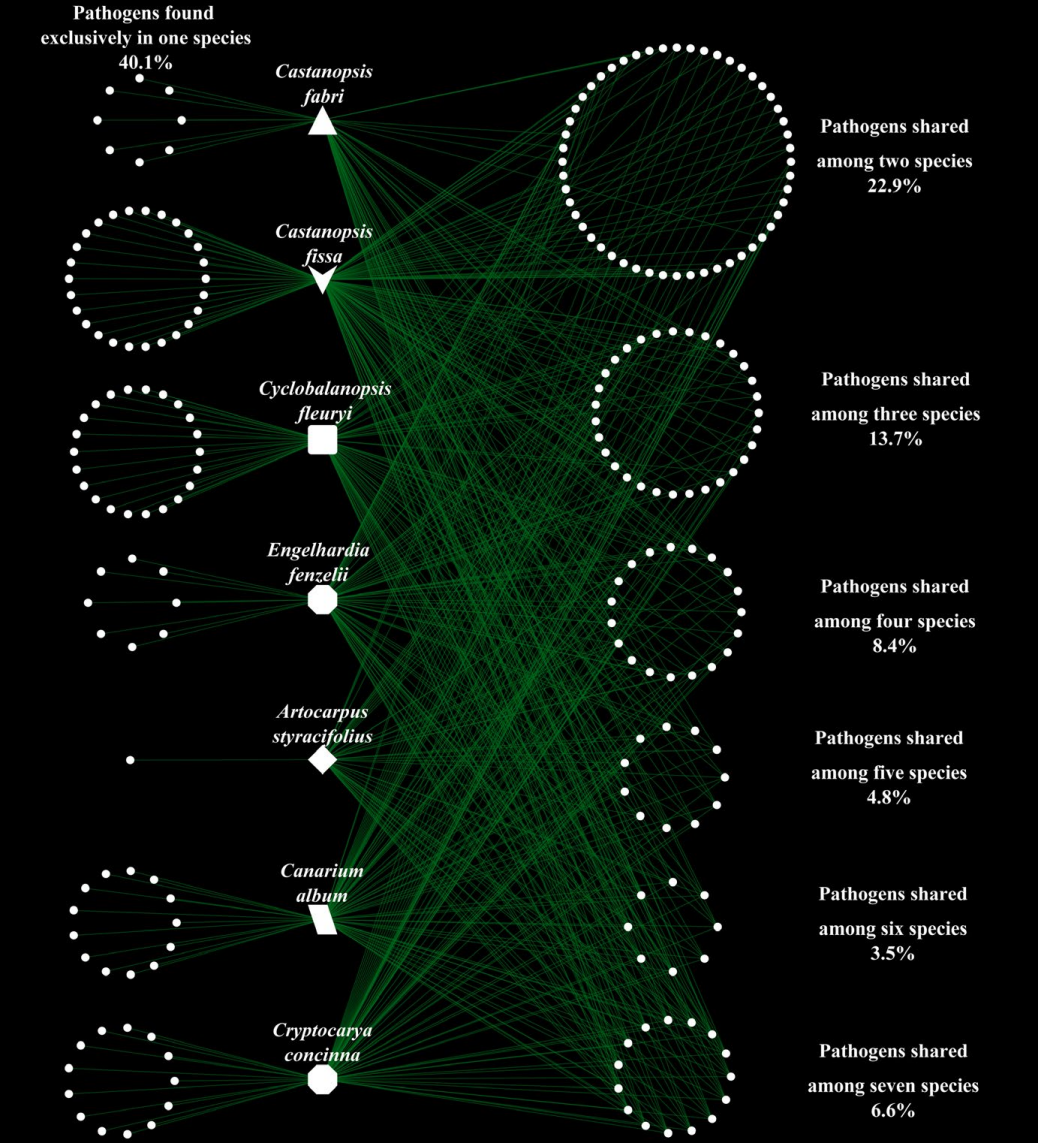
The phytopathogenic operational taxonomic unit (OTU) network map showing the interactions of the pathogenic OTUs among all the samples from different plant species. Each point represents one independent pathogenic OTU. Pathogenic OTUs on the left were considered host‐specific pathogens, while OTUs on the right were regarded as generalist pathogens

We mapped phytopathogenic OTUs shared among different focal plant species as well as OTUs unique to each plant (Figure 4) and found that 40.1% of pathogenic OTUs were detected exclusively in association with only one species, and hence were considered to be host‐specific pathogens.

**Figure 5:**
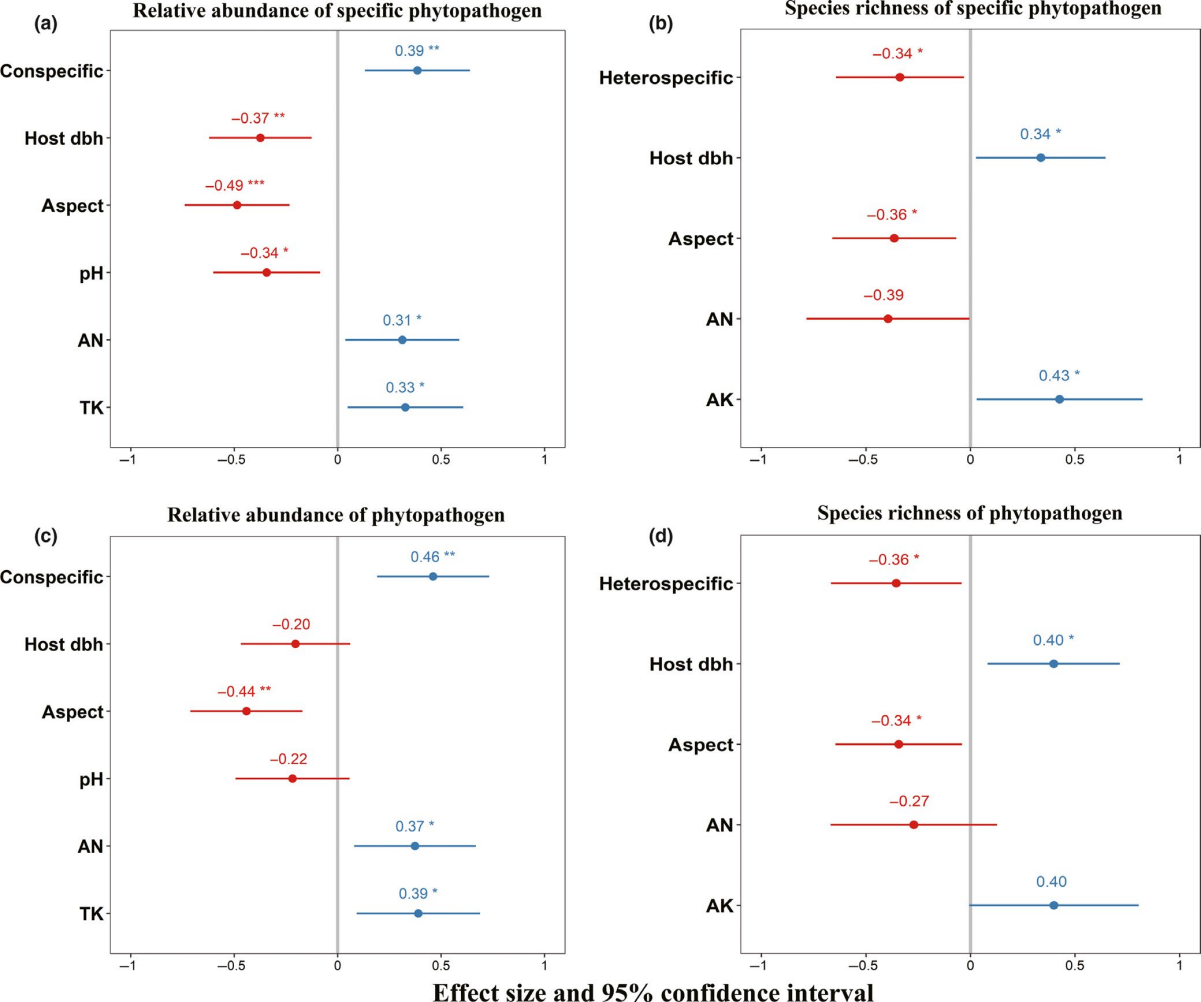
Estimated effects (mean ± standard error, *SE*) of variables on (a) relative abundance of host‐specific pathogen; (b) species richness of host‐specific pathogen; (c) relative abundance of phytopathogen (specialist and generalist); (d) species richness of phytopathogen (specialist and generalist) by the best‐fit models. ^***^<.05, ^**^<.01; ^*^<.05. Each plot shows standardized coefficient estimates for generalized linear models (GLM), representing effect size and direction for each trait with error bars indicating a 95% confidence interval in the estimate

From generalized linear model analysis, we determined that the conspecific neighbor density was significantly positively related to relative abundance of both host‐specific pathogens (estimate = 0.385, *p* = .006; Figure 5a) and phytopathogenic fungi which contained specialist and generalist pathogens (estimate = 0.461, *p* = .002; Figure 5c). Meanwhile, heterospecific neighbor density was significantly negatively associated with species richness of specialist pathogens (estimate = −0.338, *p* = .039; Figure 5b) and phytopathogens (estimate = −0.355, *p* = .034; Figure 5d). That indicated higher conspecific neighbor density was more likely to increase the abundance of pathogens, especially specialist pathogens, while higher heterospecific neighbor density would reduce the diversity of host‐specific pathogen as well as total pathogens. Additionally, the dbh of the focal adult tree can strongly influence the relative abundance and species richness of specialist pathogens with opposite effect (relative abundance: estimate = −0.374, *p* = .006; species richness: estimate = 0.336, *p* = .042; Figure 5a,b), but was unconcerned to the abundance of phytopathogen (Figure 5c).

In environmental variations, aspect proved to be an important factor influencing both relative abundance and species richness of phytopathogenic community (relative abundance of specific phytopathogen: estimate = −0.487, *p* < .001; relative abundance of phytopathogen: estimate = −0.441, *p* = .003; species richness of specific phytopathogen: estimate = −0.364, *p = *.023; species richness of phytopathogen: estimate = −0.343, *p* = .034; Figure 5). AN showed a positive relationship with relative abundance of specialist pathogen (estimate = 0.312, *p* = .035; Figure 5a) as well as pathogens (estimate = 0.374, *p* = .019; Figure 5c). TK was clearly demonstrated only positively associated with relative abundance (specific phytopathogen: estimate = 0.327, *p* = .029; phytopathogen: estimate = 0.39, *p* = .016; Figure 5a,c), while AK marginally significantly associated with species richness (specific phytopathogen: estimate = 0.427, *p* = .044; phytopathogen: estimate = 0.4, *p* = .063; Figure 5b,d).

In addition, we also calculated the species richness of total fungi and found it was significantly negatively associated with phylogenetic distance of the heterospecific neighbors (estimate = 0.48, *p* = .001; Table S3), but had no relationship with either conspecific or heterospecific neighbor density.

## 4 DISCUSSION

Some recent studies found that plants and their associated fungi are generally nonrandom assemblages (John, Maarja, Daniell, Mari, & Martin, 2011; Montesinos‐Navarro, Segarra‐Moragues, Valiente‐Banuet, & Verdú, 2012). Host plants select suitable fungal symbionts with the function of helping their own growth (Kiers et al., 2011), whereas pathogens display host preference during the infection process (Konno, Iwamoto, & Seiwa, 2011). In the current study, we defined the composition of root‐associated fungal communities in seven plant species from a subtropical forest through the use of a second‐generation sequencing approach. We found that the composition of both the root‐inhabiting fungal community and the phytopathogenic fungal community distinguished between different host plants.

Taking into account the effects of host identity, plant neighbors, and environmental factors on the fungal and the phytopathogenic fungal community composition, we found that the host species had the greatest impact on fungal community composition, while neighboring plants were the most important factor for phytopathogenic fungal community, whereas environmental conditions accounted for much smaller contributions to both fungal community assemblages. This finding indicated that host identity was the most important component in shaping the composition of a root‐associated fungal community modulated by neighbor plants. In addition, the result of the network‐level specialization test also confirmed that a high level of host specialization existed in the plant–fungal community. Moreover, the significant positive correlation between host phylogenetic distance and their fungal community dissimilarity was also detected, which indicated that phylogenetically closely related host plants were more likely to support similar fungal or pathogenic fungal communities.

All of this evidence provided a hypothesis for the host specificity of the root‐associated fungal or phytopathogenic fungal community networks, as a unique fungal community shaped by focal host identity, which is in agreement with the findings of a previous study described by Mommer et al. (2018).

In a natural ecosystem, the composition and diversity of a fungal community, especially a pathogenic fungal community, have important consequences for plant fitness and community dynamics (Benítez et al., 2013; Bever et al., 2015; Bradley et al., 2008). However, fungal community composition has been shown to be influenced by a range of biotic and abiotic factors such as soil type, season climate, and plant identity (Glinka & Hawkes, 2014; Paul, 2014; Wardle, 2006). A considerable number of papers have reported that environmental heterogeneity can affect plant growth (Buckland‐Nicks, Heim, & Lundholm, 2016) and fungal distribution (Lindahl & Olsson, 2004), whereas other studies have found that neighboring plants can largely affect the distribution of mycorrhizal fungi as a result of changing soil nutrient levels and enzyme activities (Chen et al., 2018).

Our study included field survey parameters such as environmental factors, soil properties, and the density and phylogenetic distance of neighboring species, and we analyzed the effects of these elements on the change of fungal communities, as well as on the relative abundance and species richness of phytopathogenic fungi, especially host‐specific pathogens. According to our results, the phylogenetic distance of neighboring trees has a significant impact on fungal community assemblages and the phytopathogenic fungal community, implying that this variable was an important factor in structuring host root‐associated fungal communities. In addition, conspecific neighbor density was significantly positively related to the relative abundance of host‐specific pathogens or pathogens, whereas the heterospecific neighbor density had a negative effect on the species richness of pathogens, as well as specialist pathogens. This finding revealed that conspecific neighbors attract large number of pathogens, especially host‐specific pathogens, whereas the heterospecific neighbors reduce their range.

It is worth mentioning that the diameter at breast height (dbh) of focal adult trees had a negative impact on relative abundance but a positive effect on species richness of host‐specific pathogens. The reason for this may be that, at different life stages, the host tree has different probabilities of being infected by a host‐specific pathogen. As some recent research has demonstrated, the rhizosphere microbial community is affected by plant development (Chaparro, Badri, & Vivanco, 2014), with host individuals at different host developmental stage exhibiting different responses to pathogens (Develey‐Rivière & Galiana, 2007). It is reasonable to assume that a younger tree, with a smaller dbh, would have a greater probability of being infected by a host‐specific pathogen as a result of its lower disease resistance, leading to the multiplication of the host‐specific pathogen and subsequent infection of the focal plants’ seedlings. The mature, more resistant tree, with a bigger dbh, may result in a greater species richness, with a larger number of pathogenic species, rather than an increase in the abundance of a specific pathogen. Previous studies have demonstrated that conspecific negative density dependence is strongest at tree species' early life stages (Zhu et al., 2018), which supported our speculation.

In a natural forest, the plant combination is much more complicated, as multiple hosts coexist, which means adjacent plants are important regulators of the root‐associated fungal community on a specific plant host. A number of earlier laboratory and field studies had indicated that the plant neighborhood can affect the composition of the arbuscular mycorrhizal (Hausmann & Hawkes, 2009; Mummey, Rillig, & Holben, 2005) and ectomycorrhizal fungal communities (Hubert & Gehring, 2008). On the other hand, there have been relatively few studies analyzing the mechanism of the neighboring plant effect on the pathogenic fungal community. The current study focused on this little‐studied relationship and found that the phylogenetic relatedness of the adjacent plants had a significant effect on the composition of the pathogenic fungal community on the focal species seedlings.

The mechanism underlying these relationships may involve neighboring vegetation adjusting the specific plant–pathogen interaction and consequently influencing the host specificity of the pathogen to some degree. High conspecific neighbor density would enrich the abundance of a host‐specific pathogen, and closely phylogenetically related neighbors would also tend to favor host‐preferred pathogen. Phylogenetically closely related neighbors would act as abundant inoculum sources, which could change the composition of the fungal community and cause a major shift in the fungal community in the root. Therefore, the presence of heterospecific neighbors would decrease the probability of a target seedling being colonized by a host‐specific pathogen. Consequently, the host‐specific pathogenic fungal community of one focal plant is strongly influenced by its surrounding phylogenetically related neighbors. Our study contributed to the broader perspective view that fungal communities are influenced by the presence of phylogenetically related neighboring plants and distinguished the important effects on the fungal community caused by the focal host plant and its neighbors at a local scale, findings which should help us understand the core status of neighboring plants in plant–pathogen feedback. In the future, more accurate and controllable experiments should be carried out at the glasshouse or growth room level to test this hypothesis.

From the perspective of the pathogen, an initial host‐specific pathogen aggregated in the soil around parent trees induces negative plant–soil feedback and encourages the initial establishment of heterospecific plants (Connell, 1971; Janzen, 1970), and the host‐specific interaction between plant and pathogen would persist for a long time. In return, abundant heterospecific neighboring plants distantly related to the focal plant can regulate the fungal community composition, by reducing the relative proportion of the host‐specific pathogen and decreasing the probability that a focal plant will be colonized, enhancing the survival rate of conspecific seedlings. Consequently, this continuous interaction between plants and pathogens promotes the coexistence of a large population of different tree species.

## CONFLICT OF INTEREST

None declared.

## AUTHOR CONTRIBUTIONS

KC and SY designed the study. KC conducted the experiments. KC performed statistical analyses and wrote the first draft of the manuscript, and SY contributed to revisions.

## Data Availability

The sequences of the ITS regions of partial isolates were submitted to NCBI with accession numbers SUB5647260. The data supporting this article are available from Dryad: https://doi.org/10.5061/dryad.w9ghx3fkc
