# Supplementary material for: Neighboring trees regulate the root‐associated pathogenic fungi on the host plant in a subtropical forest
Source: Ecol Evol. 2020 Apr 23;10(9):3932–43. doi: 10.1002/ece3.6094 (PMC7244890; doi:10.1002/ece3.6094)
Supplement: Supplementary file 1 [file ECE3-10-3932-s001.docx]

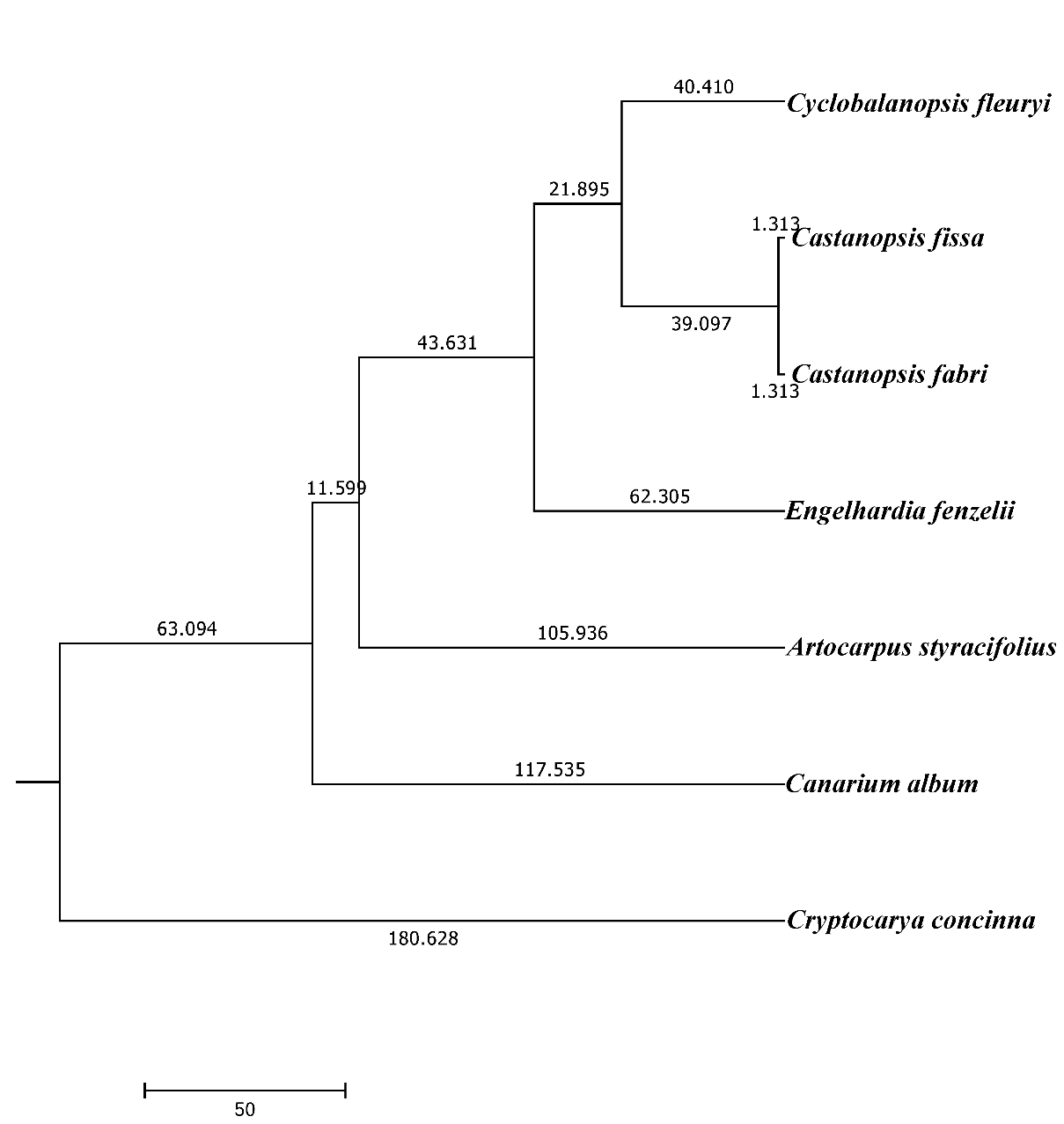


**Figure S1** Phylogenetic relationships among seven target plant species in our study using Phylocom (Webb et al. 2008) baseing on the APG III.


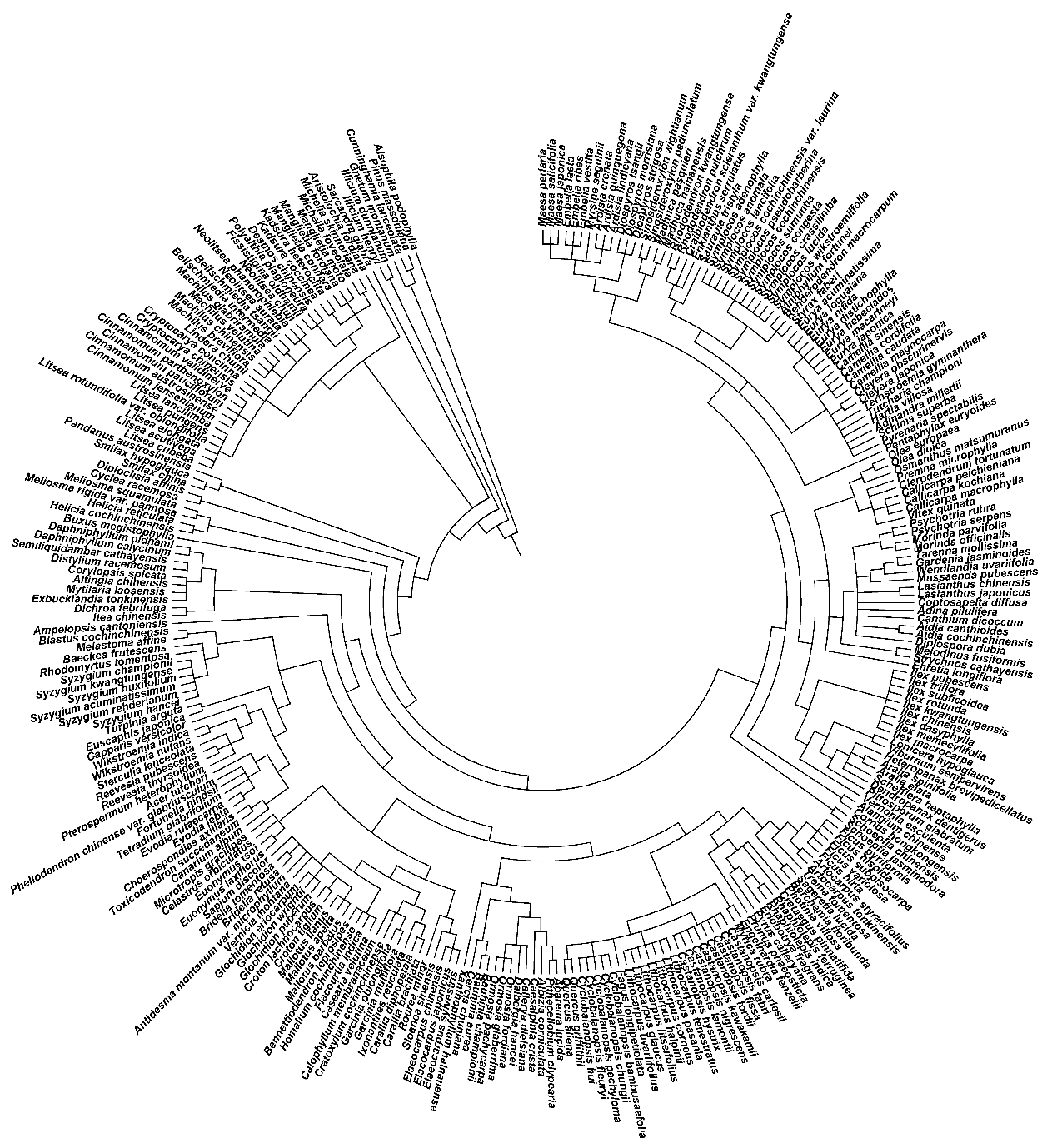


**Figure S2** The phylogenetic tree constructed based on the APG III of the 284 species in our study.
