# Supplementary material for: Neighboring trees regulate the root‐associated pathogenic fungi on the host plant in a subtropical forest
Source: Ecol Evol. 2020 Apr 23;10(9):3932–43. doi: 10.1002/ece3.6094 (PMC7244890; doi:10.1002/ece3.6094)
Supplement: Supplementary file 4 [file ECE3-10-3932-s004.docx]

**TABLE S1** Result of the network-level specialization index H2’ and t-test with null model.

|  | Observed H^2^ | Null model H^2^ | t | P |
| --- | --- | --- | --- | --- |
| Fungi | 0.534 | 0.007 | -19282.35 | **< 0.001** |
| Phytopathogenic fungi | 0.55 | 0.013 | -2451.91 | **< 0.001** |

**TABLE S2** Permutational multivariate analysis of variance (PERMANOVA) with 999 permutations showing the effects of phylogenetically distinct neighbors and habitat variation on fungal community composition.

| Variable | Fungi | | | Phytopathogenic fungi | | |
| --- | --- | --- | --- | --- | --- | --- |
|  | F.Model | R^2^ | Pr (>F) | F.Model | R^2^ | Pr (>F) |
| Phylogenetic distanc | **2.028** | **0.053** | **0.007** | **1.5** | **0.042** | **0.038** |
| Conspecific density | 1.049 | 0.027 | 0.338 | 0.841 | 0.023 | 0.723 |
| Heterospecific density | 1.281 | 0.033 | 0.11 | 0.809 | 0.023 | 0.758 |
| Host dbh | 1.204 | 0.032 | 0.198 | 1.163 | 0.032 | 0.259 |
| openness | **2.342** | **0.061** | **0.003** | **1.672** | **0.047** | **0.028** |
| slope | 1.328 | 0.035 | 0.092 | 0.833 | 0.023 | 0.728 |
| aspect | 0.897 | 0.023 | 0.603 | 1.046 | 0.029 | 0.365 |
| pH | **1.5** | **0.039** | **0.049** | **1.479** | **0.041** | **0.068** |
| OC | 0.972 | 0.025 | 0.487 | 1.153 | 0.032 | 0.279 |
| TN | **1.691** | **0.044** | **0.014** | 1.358 | 0.038 | 0.107 |
| TP | 1.303 | 0.034 | 0.102 | 1.007 | 0.028 | 0.45 |
| TK | 1.173 | 0.031 | 0.198 | 1.249 | 0.035 | 0.176 |
| AN | 1.19 | 0.031 | 0.177 | 1.085 | 0.03 | 0.337 |
| OP | 0.587 | 0.015 | 0.986 | 0.948 | 0.026 | 0.527 |
| AK | 0.719 | 0.019 | 0.921 | 0.737 | 0.021 | 0.852 |

**TABLE S3** Coefficient estimates, standard errors, *t-*value and *p*-values of generalized linear models of best model for a) relative abundance of host-specific pathogen, b) species richness of host-specific pathogen, c) relative abundance of pathogen, d) species richness of pathogen, and e) species richness of all fungi.

| **Variation** | Estimate | Standard errors | t value | P-values |
| --- | --- | --- | --- | --- |
| **a) Relative abundance of specific pathogen** | | | | |
| Host DBH | -0.374 | 0.126 | -2.959 | **0.006** |
| Conspecific density | 0.385 | 0.129 | 2.978 | **0.006** |
| Aspect | -0.487 | 0.129 | -3.762 | **< 0.001** |
| pH | -0.344 | 0.132 | -2.607 | **0.014** |
| AN | 0.312 | 0.141 | 2.221 | **0.035** |
| TK | 0.327 | 0.142 | 2.295 | **0.029** |
| **b) Species richness of specific pathogen** | | | | |
| Host DBH | 0.336 | 0.158 | 2.127 | **0.042** |
| Heterospecific density | -0.338 | 0.156 | -2.16 | **0.039** |
| Aspect | -0.364 | 0.151 | -2.408 | **0.023** |
| AN | -0.395 | 0.199 | -1.983 | 0.057 |
| AK | 0.427 | 0.203 | 2.106 | **0.044** |
| **c) Relative abundance of pathogen** | | | | |
| Host DBH | -0.204 | 0.135 | -1.51 | 0.142 |
| Conspecific density | 0.461 | 0.138 | 3.339 | **0.002** |
| Aspect | -0.441 | 0.138 | -3.194 | **0.003** |
| pH | -0.218 | 0.141 | -1.548 | 0.133 |
| AN | 0.374 | 0.15 | 2.49 | **0.019** |
| TK | 0.39 | 0.152 | 2.565 | **0.016** |
| **d) Species richness of pathogen** |  |  |  |  |
| Host DBH | 0.398 | 0.161 | 2.467 | **0.02** |
| Heterospecific density | -0.355 | 0.16 | -2.227 | **0.034** |
| Aspect | -0.343 | 0.155 | -2.222 | **0.034** |
| AN | -0.271 | 0.203 | -1.335 | 0.192 |
| AK | 0.4 | 0.207 | 1.93 | 0.063 |
| **e) Species richness of all fungi** | | | | |
| Host DBH | 0.209 | 0.132 | 1.582 | 0.124 |
| Phylogenetic distance | -0.48 | 0.137 | -3.497 | **0.001** |
| Aspect | -0.308 | 0.137 | -2.251 | **0.032** |
